# Physicochemical properties, sensory characteristics, and antioxidant activity of the goat milk yogurt probiotic *Pediococcus acidilactici* BK01 on the addition of red ginger (*Zingiber officinale* var. *rubrum rhizoma*)

**DOI:** 10.14202/vetworld.2022.757-764

**Published:** 2022-03-29

**Authors:** Sri Melia, Indri Juliyarsi, Yulianti Fitri Kurnia

**Affiliations:** Department of Animal Science, Universitas Andalas, Padang, 25163 West Sumatra, Indonesia

**Keywords:** color, *Pediococcus acidilactici BK01*, probiotic, red ginger, yogurt

## Abstract

**Background and Aim:**

Yogurt contains beneficial probiotics. Addition of red ginger to yogurt as an antioxidant source becomes a way to improve the flavor and functional properties of yogurt. This study aimed to examine yogurt processing and the effect of adding red ginger *(Zingiber officinale* var*. rubrum rhizom*a), as an antioxidant source, on *Pediococcus acidilactic*i BK01. It sought to observe the physicochemical and sensory qualities during storage (4°C).

**Materials and Methods:**

Goat milk was obtained from local farmers in Lubuk Minturun, Padang, West Sumatra, Indonesia. The yogurt was divided into two factors. Factor A was supplemented with red ginger in various concentrations: A (0% – as control), B (1%), C (2%), and D (3%). On the other hand, Factor B was subjected to variations in storage time: A (1 day), B (15 days), and C (30 days). Each treatment was conducted in triplicate. Physicochemical properties (pH, titratable acidity [TTA], and proximate analysis), sensory characteristics, and antioxidant activity (2,2-diphenyl-1-picrylhydrazyl radical scavenging capacity) were measured using the standard tests. The data were analyzed through analyzing multivariate (analysis of variance) supported by Duncan’s multiple range test.

**Results:**

The addition of red ginger juice increased the antioxidant activity, TTA, and water holding capacity (WHC) (p<0.05), while syneresis was significantly decreased; however, it had no effect on the total lactic acid bacteria. At the end of this research (day 30), the yogurt was still suitable for consumption, with the following composition: Antioxidant activity 48.39%, pH 4.3, TTA 1.716, water content 80%, protein 3%, fat 3%, syneresis 28%, WHC 63%, and total lactic acid bacteria 89×10^8^ colony-forming units/mL. Furthermore, yogurt supplemented with red ginger changed its color into red blush.

**Conclusion:**

Yogurt red ginger juice is recommended as a functional drink, as it contains probiotics *P. acidilactici* BK01 and antioxidants to support human health. The addition of up to 3% ginger juice and a storage period of 30 days are still favored by the panelists and meet the quality standard of yogurt. We have not conducted the study on active compounds so, further research could be conducted on the components of the active compounds found in red ginger yogurt.

## Introduction

Cow milk and goat milk can both be used to make yogurt. Goat milk is now increasingly being used in the milk fermentation process [1]. Goat milk has several advantages, including a higher nutritional composition (high calcium and inorganic phosphorus) and volatile fatty acids (caproic, caprylic, and capris) that are responsible for the specific aroma and flavor [2]. Furthermore, since goat milk is easier to digest than cow milk, it is also safe for consumption by lactose-intolerant people [3 ], potentially produced preferably as yogurt. Yogurt is a milk product that is popular all over the world. At present, yogurt production is characterized by several varieties and modifications. Certain approaches modify the composition of cow milk to make it similar to that of goat milk. In contrast, others change the probiotic (lactic acid bacteria) as the starter with extra fruit juice. During milk fermentation, lactic acid bacteria, particularly probiotic bacteria that confer numerous health benefits on the digestive tract, can inhibit the growth of pathogenic bacteria such as *Escherichia coli, Staphylococcus aureus*, and *Listeria monocytogenes* [4,5]. When consumed regularly, fermented milk products such as yogurt can help strengthen the body’s immune system.

Recently, several types of fermented milk have been flavored with various fruits – either whole fruit, fruit juice, or fruit extract – to improve its taste and functional properties. Yogurt has been flavored, for example, with pineapple and carrots [6]. However, yogurt research and development is not focused on the addition of spices and herbs, such as red ginger. Red ginger (*Zingiber officinale* var. *rubrum rhizoma*) is widely used in food and beverages because of its hypolipidemic, anti-emetic, anti-viral, anti-motion, and anti-inflammatory properties [7,8]. Furthermore, red ginger contains about 40% dry weight of starch [9]. Starch is a source of energy and can be used as a thickening agent in the yogurt-making process [10]. Since the starch in red ginger can improve the texture of the yogurt, in this research, we leveraged this property to restore yogurt thickness by adding red ginger before the fermentation process.

This study aimed to observe the quality of goat milk yogurt supplemented by red ginger right before the fermentation process by *Pediococcus acidilactici* BK01 as a starter during cold storage (4°C).

## Materials and Methods

### Ethical approval

No ethical approval was required for this study because no live animals were used in this study.

### Study period and location

The study was conducted from March to July 2021 at the Animal Products Technology Laboratory, Department of Animal Husbandry, Universitas Andalas in Padang, Indonesia.

### Yogurt starter and red ginger juice

The starters used in this study, *Streptococcus thermophilus* and *Lactobacillus fermentum* L23, were obtained from Animal Product Technology Laboratory, Animal Science Faculty, Universitas Andalas. In addition, *Pediococcus* BK01 was isolated from Bekasam, South Sumatera [11]. Goat milk was obtained from local farmers in Lubuk Minturun, Padang, West Sumatra, Indonesia. Red ginger was obtained from the local market in Padang, West Sumatera, Indonesia. Ginger was grated and filtered to make red ginger juice, which was then pasteurized at 70°C for 10 min.

### Manufacture of yogurts

The research used three combinations of bacteria: *S. thermophilus, L. fermentum* L23, and *P. acidilactici* BK01. Raw milk of crossbreed Etawa goat from local farmers in Padang, West Sumatera, Indonesia, was used. Milk was pasteurized in a water bath at 70°C for 30 min before immediately cooling it at 37°C. Up to 5% (v/v) yogurt starter was added to the milk; further, red ginger juice of 0%, 1%, 2%, and 3% was added, and then, it was incubated at 37°C for 12 h or until the pH reached 4.5. Following that, the yogurt was stored at a low temperature of 4°C for 1 day, 15 days, or 30 days depending on the treatment employed to measure its physicochemical and microbiological properties.

### Physicochemical analyses

#### pH, titratable acidity (TTA), and proximate analysis

After pH 4.0 and pH 7.0 standards, the pH was measured using a pH meter (Hanna Instruments, Romania). To measure the TTA, 10 mL yogurt was mixed with a phenolphthalein indicator titrated with 0.1 N NaOH until the pink color was displayed. The Association of Official Analytical Chemists [12] no. 4 method was used to analyze the moisture, protein, and fat content. After 1 day, 15 days, and 30 days of storage, all the parameters were tested with three replications.

### 2,2-Diphenyl-1-picrylhydrazyl (DPPH) radical scavenging capacity of yogurt

In 375 μL ethanol (99%) and 125 μL DPPH solution (0.02% in ethanol), sample volumes of 500 μL were supplemented as a free radical source at different concentrations. (25 μg/mL, 100 μg/mL, 75 μg/mL, 50 μg/mL, and 25 μg/mL). At room temperature (25°C) in the dark, the mixture was twisted and incubated for 60 min. A spectrophotometer (ultraviolet [UV] mini 1240, UV/visible spectrophotometer, SHIMADZU, Kyoto, Japan) was used to measure the radical scavenging capacity and control absorbance at 517 nm. DPPH has a 517 nm absorption band, which is eliminated when anti-radical compounds are reduced. The lower absorption level indicates that the reaction mixture likely possesses increased radical scavenging activity. The DPPH radical scavenging capacity is calculated as follows:


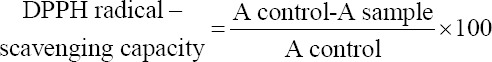


Control is the absorption of the control reaction (that includes all but the sample reagents) and a sample is the sample absorption (with the DPPH solution).

### Color of the yogurt samples

The yogurt color was measured with a colorimeter (Lab Scan II, Hunter Associate Laboratory Inc., Reston, VA, USA). In the glass refract cup of the light pore, 44.45 mm of the yogurt samples were placed. Data were recorded in CIE (The Commission Internationale de l’Eclairage) L*/lightness (L*) values, chromium (C) (a*^2^+b*^2^)^1/2^ values, and Tan^-1^ (b*/a*) Hue angle (h°) values for saturation and color shade. Water holding capacity (WHC)

The WHC was measured for 20 g of the yogurt sample (y) for 20 min at 4500×g, 4°C, and centrifuged temperatures (centrifuge 5417R). The whey expelled (WE) was separated and weighed [13]. WHC was measured with three replications on 1 day, 15 days, and 30 days of storage.

WHC can be measured using this formulation:

WHC (%)=100 (Y-WE)/Y.

### Syneresis

A centrifugation method was used to determine syneresis. A glass rod was removed manually for a set of yogurt. A conical tube (Becton Dickinson Laboratory, NJ, USA) was transferred to a 50 mL Blue max ™ polypropylene tube (corning, USA), which was left to stabilize at 4°C for 2 h. Samples were then centrifuged at 3313 g (Sorvall RT 7; Sorvall Production L.P. USA) for 15 min. The syneresis was expressed through the initial weight of gel as the percentage of whey separated from the gel [14]. Syneresis was measured after 1 day, 15 days, and 30 days.

### Sensory evaluation

The yogurt samples were mixed with red ginger juice as A1 (0%), A2 (1%), A3 (2%), and A4 (3%). Organoleptic assessment of taste, aroma, and texture was carried out by 30 staff members of the Animal Science Department who had consumed yogurt before and were familiar with its sensory properties. A 5-point hedonic scale was utilized in this study (1=Dislike extremely; 2=Dislike slightly; 3=Neither like nor dislike; 4=Like slightly; and 5=Like extremely).

### Calculation of total lactic acid bacteria

The number of De Man, Rogosa, and Sharpe (MRS) colonies of lactic acid bacteria was calculated to be 10^-7^ using MRS broth medium (Merck, Germany) and it was incubated at 37°C for 48 h after serial dilution. Total lactic acid bacteria were calculated in colony-forming units (CFUs)/mL on day 1, day 15, and day 30 [15].

### Statistical analysis

The yogurt was arranged into two factors. Factor A was supplemented with red ginger in various concentrations: A (0% – as control), B (1%), C (2%), and D (3%). On the other hand, Factor B was subjected to variations in storage time: A (1 day), B (15 days), and C (30 days). Each treatment was conducted in triplicate. The data were analyzed using analyzing multivariate (analysis of variance) supported by Duncan’s multiple range test. The expressed values are the mean±standard deviation from the 3-fold measurements (n=3).

## Results and Discussion

### Physicochemical analysis

#### pH of yogurt

**Figure-1:**
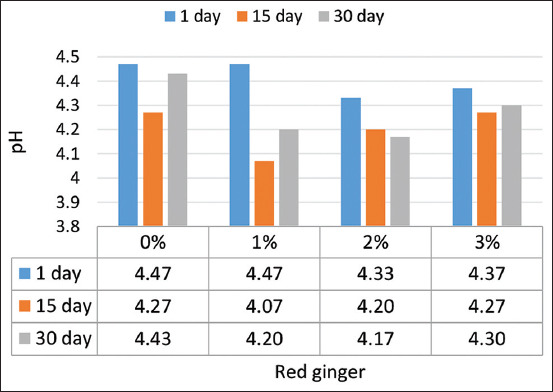
pH of red ginger juice yogurt during storage.

A significant reduction (p=0.0270) in pH was observed for the yogurt with red ginger juice. The pH of the yogurt was reduced to 4.31 on the addition of 3.0% ginger (Figure-1). The decrease in the pH of yogurt with an increase in the added red ginger can be attributed to the reduction of the yogurt by the acidic red ginger juice. The pH of this yogurt decreased significantly until 35 days of storage, when it was noted to be 4.28. The pH reduced from 4.46 to 4.42 on the 1^st^ day of storage and slightly increased from 4.22 to 4.26 after 28 days; similar results were also observed by Madhubasani *et al*. [16]. Cinnamon-added yogurts had a pH between 3.98 and 4.20 and a TTA between 0.74 and 1.97 [17]. The pH was found to decrease from 4.62 (control) to 4.44 on the addition of 2.5% ginger powder [18].

Lactose-producing organic acids cause a reduction in pH. Lactose was converted into glucose and galactose and divided into lactic acid by lactose fermentation [19]. The culture’s metabolic activity decreases the pH during stocking [20]. The reduction in pH causes the casein micelles to reach an isoelectric point during fermentation (pH 4.6); it fosters the solubilization of colloidal calcium, enhances hydrophobia interactions, and thus causes casein micelles to form three-dimensional compounds. Lucey [21] and Phadungath [22] also stated that casein micelles play an essential role in the coagulation of milk acid.

#### TTA

**Figure-2:**
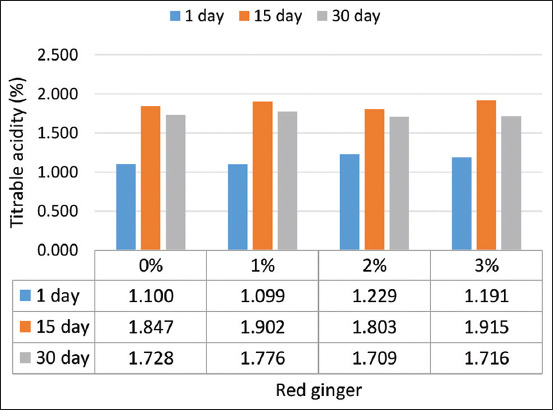
Titratable acidity of red ginger juice yogurt during storage.

The addition of red ginger juice to yogurt significantly (p=0.0455) increased the TTA from 1.607% to 3% (Figure-2). The increase of TTA yogurt was in line with the decrease in the yogurt pH value with the increase in the red ginger juice added. Similarly, the storage time increased substantially (p<0.05; 1.732%) to 30 days. The TTA of the yogurt increased during storage [23]. Storage of rice yogurt for 21 days following the addition of rice milk increases the TTA value [24,25] and reduces the pH on day 28. The post-acidification of yogurt during cold storage is responsible for *Lactobacillus delbrueckii* subsp. *bulgaricus* and *S. thermophilus* [26].

#### The composition of red ginger yogurt

After the addition of 3% red ginger juice, the water content of the yogurt was significantly reduced (p=0.0335). (Table-1). In this study, it was observed that the lower the water content, the higher the protein content of the yogurt produced. The present results reveal 87.49% decrease in the water content compared to the research results of Vianna *et al*. [27].

**Table-1:** Yogurt composition of red ginger yogurt on cold storage (4°C).

Sample	Storage time (days)			Average

	1	15	30	
Moisture				
A1 (control)	82.30	81.91	81.00	81.74^a^
A2 (1%)	81.01	79.83	80.45	80.43^b^
A3 (2%)	80.42	79.16	80.53	80.04^c^
A4 (3%)	79.71	78.64	80.93	79.76^d^
Average	80.86	79.89	80.73	(p=0.0335)
Protein				
A1 (0% red ginger juice)	3.31	3.27	3.33	3.30^a^
A2 (1% red ginger juice	3.43	3.20	3.33	3.31^b^
A3 (2% red ginger juice)	3.26	3.45	3.25	3.32^c^
A4 (3% red ginger juice)	3.24	3.27	3.45	3.32^c^
Average	3.31	3.30	3.33	(p=0.046)
Fat				
A1 (0% red ginger juice)	3.33	3.40	3.36	3.36
A2 (1% red ginger juice	3.42	3.35	3.21	3.33
A3 (2% red ginger juice)	3.33	3.29	3.47	3.36
A4 (3% red ginger juice)	3.36	3.38	3.27	3.34
Average	3.36	3.35	3.33	(p=0.4420)

Data without superscripts indicate no significant differences (p>0.05)

After the addition of red ginger juice, the protein content of the red ginger juice increased (p=0.046) considerably (Table-1). The red ginger jug yogurt content is virtually the same as the 3.54% and [23], which was 2.53-3.43%, but higher than the protein level [26] and, that is, 3.16%. The low water content was also a result of the influence of the red ginger juice. Protein helps improve the WHC of the yogurt [18]. Rosell *et al*. [28] stated that higher protein content may result in higher WHC, thus preventing water loss through the attraction and binding of water during the processing of food.

No significant change was observed in the fat content of the red ginger yogurt (p=0.4420) in terms of ginger savory and time storage (Table-1). The fat content of red ginger juice yogurt was almost the same as that found in a study where the cinnamon yogurt was stored at 4°C for 21 days at fats 2.90-3.25% [17], which is in rice milk of yogurt 3.44% [24].

#### DPPH radical scavenging capacity of yogurt

**Figure-3:**
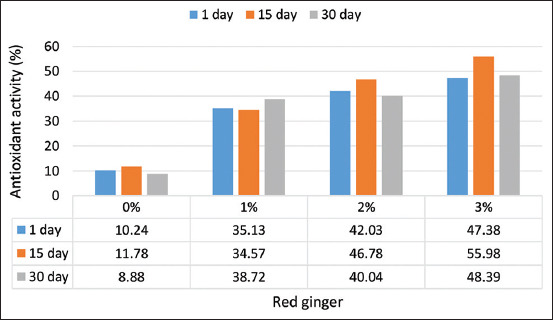
Antioxidant activity of red ginger juice yogurt during storage.

The results showed that adding red ginger juice to yogurt significantly increased antioxidant activity (p=0.0102). Up to 3% of red ginger juice increased the antioxidant activity up to 55.98% on 15 days of storage and up to 48.39% after 30 days (Figure-3). The results indicate that the red ginger juice used in this study had an antioxidant activity of 77.059%. The increased addition of red ginger juice to yogurt can increase the antioxidant activity. Research on yogurt with 1% ginger powder [18] showed that the antioxidant activity increased to 95.56% after 21 days of storage. Larasati *et al*. [29] noted that more than 0.5% antioxidant activity of yogurt with red ginger extract was 71.60%.

#### Color of yogurt

The addition of up to 3% red ginger juice decreased the L value significantly (p=0.0421) from 80.65% (control) to 77.11% (Table-2). On the addition of 2.5% ginger powder to yogurt, the L value decreased from 91.58 to 82.32 [18]. The L value of the present study was lower than that of Akgun *et al*. [30], where the L value of buffalo milk yogurt ranges from 84 to 86%. The present study used goat milk, which has lower fat content than buffalo milk, thus affecting the L value.

**Table-2:** The value of (L, a*, and b*) red ginger juice yogurt.

Sample	Storage time at 4°C			Average

	1	15	30	
Lightness (L)				
A1 (0% red ginger juice)	82.06	82.92	76.98	80.65^a^
A2 (1% red ginger juice	81.74	81.87	74.10	79.24^b^
A3 (2% red ginger juice)	80.13	83.80	75.19	79.71^c^
A4 (3% red ginger juice)	75.78	80.50	75.04	77.11^d^
Average	79.93^a^	82.27^a^	75.33^b^	(p=0.0421)
Greenness (a*)				
A1 (0% red ginger juice)	−2.99	−2.99	−2.92	−2.97^a^
A2 (1% red ginger juice	−3.17	−3.17	−2.52	−2.95^b^
A3 (2% red ginger juice)	−2.70	−2.93	−2.82	−2.82^b^
A4 (3% red ginger juice)	−2.63	−2.58	−2.83	−2.68^c^
Average	−2.87	−2.92	−2.77	(p=0.0233)
Yellowness (b*)				
A1 (0% red ginger juice)	2.12	3.42	3.58	3.04^a^
A2 (1% red ginger juice	3.55	3.37	3.02	3.31^a^
A3 (2% red ginger juice)	3.95	3.31	3.15	3.47^a^
A4 (3% red ginger juice)	4.62	3.44	4.18	4.08^b^
Average	3.56	3.38	3.48	(p=0.0104)

Data without superscripts indicate no significant differences (p>0.05)

The addition of 3% red ginger juice to yogurt during storage significantly increased (p=0.0233) a* value and (p=0.0104) b* values. It was influenced by the dark and yellowish color of the red ginger juice. The a* value of the present study was almost similar to that noted in Akgun *et al*. [30]. Yogurt with the addition of ginger powder had an a* value of −5.41 (control), which increased with the addition of 2.5% ginger to −0.045 [18]. The control b* value was 15.60, while with the addition of 2.5% ginger, it increased to 21.23.

#### WHC

**Figure-4:**
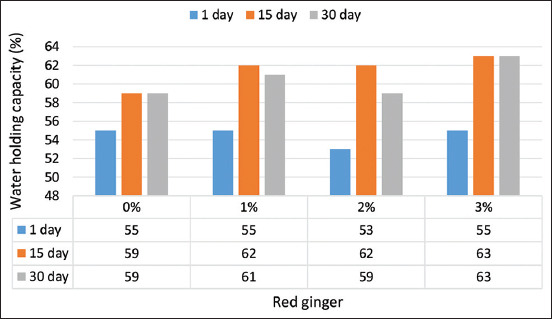
Water holding capacity yogurt red ginger juice.

The addition of up to 3% red ginger juice resulted in a significant increase (p=0.0373) of the yogurt’s WHC (Figure-4). Red ginger contains starch that can bind water during yogurt processing; Ibrahim and Khalifah [9] observed that ginger contained up to 40% (dry basis) starch. Starch can form a gel and bind water during the production of yogurt, as highlighted by Lobato-Calleros *et al*. [31]. Furthermore, Felfoul *et al*. [18] stated that ginger powder stabilizes yogurt to prevent whey separation. Protein also contributes to the increase in the WHC of yogurt. In the present study, the addition of red ginger concentrations to yogurt caused an increase in its protein content, thus increasing its WHC. According to Rosell *et al*. [28], higher protein content can result in improved WHC. A greater WHC will reduce the quantity of water lost in food processing at high temperatures.

The duration of storage had a significant effect (p<0.05) on increasing the WHC of the yogurt supplemented with red ginger juice (Figure-4). Total solids and protein content affect the WHC. A large quantity of protein in the milk may increase the yogurt gel network density and result in WHC [32]. Sodini *et al*. [33] reported that the increase in casein concentration may favor micellar interactions, resulting in decreased pore dimensions of the matrix and increased density.

#### Syneresis

**Figure-5:**
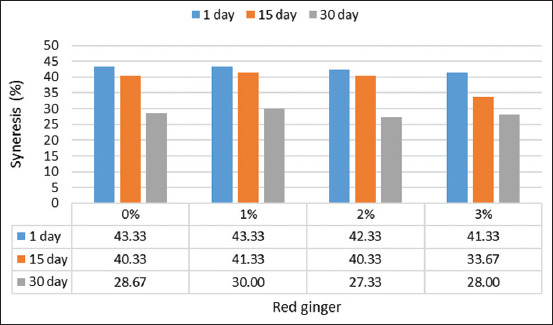
Syneresis of red ginger juice yogurt during storage.

The addition of up to 3% red ginger juice resulted in a significant change (p=0.0367) in the syneresis value of the yogurt. Yogurt with 3% red ginger juice had a syneresis value of 28% on day 30, which was lower than the 41.33% recorded on day 1 (Figure-5). This result is lower than that of Vianna *et al*. [27]. This result is lower than that of Vianna *et al*. [27], where syneresis of goat milk yogurt increased to 44.35% on the 1^st^ day of storage and to 51.18% after 28 days.

The decrease in yogurt syneresis after storage was also noted in Akgun *et al*. [30]; yogurt syneresis decreased on day 20 of storage. Yogurt with 2.5% ginger powder may reduce the syneresis to as much as 35.61%, according to Felfoul *et al*. [18]. Total solids increase because of the high starch content in ginger [34]. The presence of whey (serum) on the surface of yogurt, which can decrease the consumer’s acceptance of the product, is of significant importance in commercial manufacturing. Therefore, adding red ginger juice may prevent yogurt syneresis growth while stored. In addition, the highest syneresis rate was shown by yogurt made from goat’s milk [35].

#### Sensory evaluation

**Figure-6:**
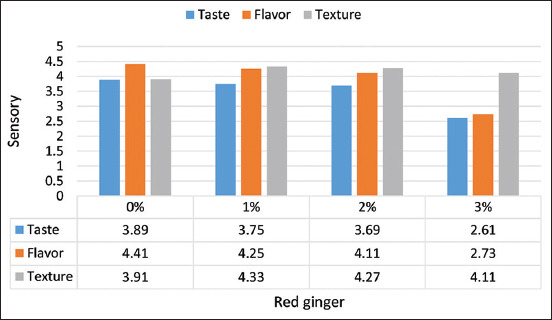
Sensory evaluation of red ginger juice yogurt during storage.

The results of the sensory evaluation of red ginger juice yogurt are illustrated in Figure-6. The addition of red ginger juice significantly affected the acceptability of the yogurt’s taste and aroma; the panelists still preferred adding 2% red ginger juice (value 4). Concerning the yogurt’s texture, the addition of red ginger juice did not affect the consistency of the yogurt, and the addition of up to 3% was still preferred. The presence of starch in red ginger produces yogurt with a more compact texture. Further, research by Felfoul *et al*. [18] noted that ginger powder acts as a stabilizer in yogurt to prevent whey separation.

#### Total lactic acid bacteria in yogurt with red ginger juice

The addition of red ginger juice to yogurt did not affect (p=0.5555) the total lactic acid bacteria count. Total lactic acid bacteria in ginger yogurt ranged from 73×10^8^ CFU/mL to 92×10^8^ CFU/mL (Table-3). In research [36], the addition of up to 4% ginger juice to yogurt was favored by the panelists, and a total lactic acid bacteria count of 1285×10^9^ CFU/mL was recorded. Rosell *et al*. [28] stated that yogurt with red ginger extract up to 0.5% reduced the total lactic acid bacteria count from 90.37×10^13^ CFU/mL (control) to 4.86×10^13^ CFU/mL.

**Table-3:** The total value of lactic acid bacteria.

Sample	Storage time at 4°C			Average
	1	15	30	
Lactic acid bacteria (×10^8^ CFU/mL)				
A1 (0% red ginger juice)	41	95	82	73
A2 (1% red ginger juice	62	177	34	91
A3 (2% red ginger juice)	64	150	61	92
A4 (3% red ginger juice)	42	157	67	89
Average (p=0.0001)	52^b^	145^a^	61^b^	

Data without superscripts indicate no significant differences (p>0.05).

Storage time had a significant effect (p=0.0001) on the total lactic acid bacteria count. The results showed that the total lactic acid bacteria in red ginger yogurt increased up to 145×10^8^ CFU/mL after 15 days of storage; however, after 30 days of storage, the total lactic acid bacteria count decreased to 61×10^8^ CFU/mL (Table-3). This is because the longer the storage, the higher the amount of organic acids produced by the lactic acid bacteria, causing a decrease in pH; the high acidity, consequently, causes its death. This follows the observation of Prasanna *et al*. [20] that a reduction in the lactic acid bacteria count of a product is closely related to a decrease in the pH value of the product due to the accumulation of organic acids produced during fermentation. In addition, a longer storage time deprives the bacteria of nutrients, resulting in its death.

The trends observed in the results of the present study of yogurt are consistent with that of Melia *et al*. [37]. Although the present research used a combination of three bacteria with added red ginger, a reduction in the total count of lactic acid bacteria was still observed during refrigerated storage. On the other hand, Melia *et a*l. [37], which used only *P. acidilactic*i BK01 colonies without any supplementation, showed the same trend, but it was still based on the criteria of functional food. Similarly, Sarwar *et al*. [38] showed that the total lactic acid bacteria count decreased during the 28-day storage at 4°C for yogurt. The probiotic microflora was reduced during storage in Vinderola and Reinheimer [39]. Loss of cell viability depends on the type of yogurt and the application of starter lactic acid bacteria.

## Conclusion

Based on the research, it can be concluded that the addition of more than 3% red ginger juice with a storage time of 30 days was still favored by the panelists and met the quality standards of yogurt. After 30 days of storage, red ginger juice yogurt had the highest antioxidant activity at 48.39%, pH 4.3, TTA 1.716, water content ±80%, protein ±3%, fat ±3%, syneresis ±28%, WHC ±63%, total lactic acid bacteria count 89×10^8^ CFU/mL, and total plate count 52×10^2^ CFU/mL. The addition of red ginger juice increased the color values of a* and b* but decreased the L value of the yogurt. The red ginger supplement gives extra function to the yogurt product. Besides being a source of antioxidants, red ginger also improves the texture of the yogurt. However, it must be noted that red ginger adds a bitter taste to the yogurt; nevertheless, up to 3% was still accepted by the panelists engaged in this study to taste the yogurt. Furthermore, yogurt with red ginger juice can be used as a functional food because it contains beneficial probiotics and antioxidants. We have not conducted the study on active compounds so, further research could be conducted on the components of the active compounds found in red ginger yogurt.

## Authors’ Contributions

SM: Carried out the research and wrote the manuscript. SM, IJ, and YFK: Processed the data. All authors read and approved the final manuscript.
